# Technological Model to Optimize the Request for Radiological Studies

**DOI:** 10.7759/cureus.92135

**Published:** 2025-09-12

**Authors:** Juan D Vásquez, Carlos A Galeano Baquero, David González, Catalina Posada Cuartas, Simón Samuel Cadavid Barrios

**Affiliations:** 1 Radiology, Clínica SOMER (Sociedad Médica Rionegro SA), Rionegro, COL; 2 Radiology, SURA (Suramericana SA), Medellín, COL; 3 Radiology, Prodiagnóstico, Medellín, COL; 4 General Practice, Universidad CES (Corporación de Estudios de la Salud), Medellín, COL

**Keywords:** abdominal pain, algorithm, decision-making, e-learning, exam requests, quality of care

## Abstract

Inappropriate requests for imaging studies are a frequent problem in clinical practice, leading to diagnostic errors, unnecessary costs, and patient dissatisfaction. These errors often arise from insufficient dissemination of clinical guidelines, limited training of referring physicians, and variability in request formats. The result is delayed diagnoses, duplication of studies, increased radiation exposure, and inefficient use of healthcare resources. To address this issue, technological tools such as diagnostic algorithms have been proposed to support physicians in selecting the most appropriate imaging tests, especially in time-sensitive conditions.

This study evaluated a diagnostic algorithm through a cross-sectional survey of 111 participants, including physicians, residents, interns, medical students, and dental professionals. The questionnaire explored perceptions of the algorithm’s clinical utility, clarity, and feasibility of integration into daily workflows. Respondents consistently highlighted its capacity to improve diagnostic accuracy, expedite decision-making, and facilitate clearer communication between physicians and radiologists. Specific strengths included its applicability to abdominal emergencies and complex scenarios such as right upper quadrant pain, jaundice, pancreatitis, and trauma.

At the same time, participants pointed out challenges, including difficulties in evaluating contrast safety, limited access to high-cost imaging, and the need for broader diagnostic coverage and personalization by age group. Despite these concerns, the algorithm was positively received overall and was recognized as a useful support tool for reducing inappropriate requests, enhancing diagnostic confidence, and ultimately improving patient care.

## Introduction

Inappropriate indications for imaging studies are a frequent problem in clinical practice, with significant implications for diagnostic accuracy, patient safety, and healthcare efficiency. Errors in test selection, patient preparation, and the consideration of comorbidities can lead to delays in diagnosis, unnecessary duplication of studies, excessive radiation exposure, and rising healthcare costs [1]. In addition, these mistakes often generate dissatisfaction among patients, who may undergo examinations that do not contribute to solving their clinical problem.

Several studies conducted in different healthcare systems have documented that unclear institutional protocols and inconsistent request formats are recurrent causes of errors, particularly in the use of contrast media and the choice of imaging modality [2]. Limited knowledge among referring physicians, insufficient dissemination of guidelines, and variations between institutions further aggravate the problem. Although societies such as the American College of Radiology and the Argentine Society of Radiology have developed evidence-based guidelines to support appropriate decision-making, their implementation remains suboptimal, especially in emergency settings where decisions must be made rapidly [3,4].

In recent years, technological tools such as computerized algorithms and digital platforms have been proposed to support physicians in the rational selection of imaging tests. These tools aim to optimize clinical decision-making, reduce variability in practice, and improve communication between physicians and radiologists. However, the real usefulness of these instruments depends on their acceptance by healthcare professionals and trainees, as well as on their adaptability to diverse clinical scenarios [5].

Based on these considerations, the present study explores the evaluation of a diagnostic algorithm designed to guide imaging requests, particularly in abdominal emergencies and complex conditions. By gathering perspectives from a diverse group of participants, the study seeks to assess its perceived utility, identify its strengths and limitations, and outline potential avenues for refinement and broader application in clinical practice [6,7].

## Materials and methods

Study design

A mixed-methods validation study was designed to evaluate the clarity, applicability, and potential integration of diagnostic imaging algorithms developed for common clinical scenarios. The validation approach combined structured presentations with a standardized survey instrument, allowing the collection of both quantitative and qualitative data. The objectives of this study were threefold: (1) to determine whether the algorithms were perceived as clear and clinically useful, (2) to explore their educational value for different groups of healthcare professionals and trainees, and (3) to assess their feasibility for integration into routine clinical workflows.

Study population and sampling

Participants were recruited through purposive sampling, which in this context referred to the intentional selection of individuals directly involved in the request, interpretation, or management of diagnostic imaging. The sample included a diverse group of stakeholders: medical students (considered trainees), interns, residents from multiple specialties, general practitioners, emergency physicians, surgeons, radiologists, and department heads. Administrative staff were also included, defined as hospital personnel responsible for coordinating radiology services and supporting clinical decision workflows (e.g., scheduling, patient logistics). In total, 111 participants from several teaching hospitals and academic centers were enrolled. All participants provided informed consent, including permission for the anonymous use of their responses in academic publications. While department heads are indeed physicians, they were analyzed separately to highlight their dual role as clinicians and service leaders.

Validation procedure

Three validation sessions were conducted, each lasting approximately one hour, and organized according to specialty group. Each session followed a standardized format: (i) Introduction: A brief overview of the study objectives and explanation of the rationale for using diagnostic algorithms in clinical decision support; (ii) Presentation of the algorithms: A step-by-step explanation of the logic, structure, and intended use of the algorithms covering scenarios such as contrast allergy, right upper quadrant pain, jaundice, acute pancreatitis, blunt and penetrating abdominal trauma, among others. Emphasis was placed on clinical clarity, decision-making flow, and points of integration with imaging guidelines; (iii) Survey administration: Participants completed a structured questionnaire assessing the clarity, clinical relevance, completeness, usability, and educational value of the algorithms. A space for open-ended comments was provided to capture qualitative feedback.

Survey instrument

The survey was delivered via a secure online form and structured in three sections: (i) Demographic data: Name (optional), professional or trainee role, academic level, and years of experience; (ii) Model evaluation: Fourteen items specifically designed to evaluate the algorithms across domains of clarity, practicality, completeness, and usability. These items were formulated by the project team and reviewed by radiology and emergency medicine experts for content validity; (iii) Open-ended feedback: A free-text field for participants to provide suggestions, perceived advantages, limitations, and recommendations.

Table 1 shows the complete survey instrument applied in this study.

**Table 1:** Survey questionnaire for validation of diagnostic imaging algorithms

Section	Question	Survey question
Demographics	1	Name (optional)
Demographics	2	Professional/trainee role
Demographics	3	Academic level
Demographics	4	Years of clinical or academic experience
Model evaluation	5	Do you consider that the algorithm for classification of contrast media allergies is clear and easy to follow?
Model evaluation	6	Does the algorithm for right upper quadrant pain provide practical steps that guide differential diagnosis?
Model evaluation	7	Does the algorithm for patients with jaundice facilitate the appropriate selection of initial imaging studies?
Model evaluation	8	Is the algorithm for acute pancreatitis useful to determine the need for additional studies and their timing?
Model Evaluation	9	Does the proposed algorithm for blunt abdominal trauma facilitate the decision between invasive and non-invasive studies?
Model evaluation	10	Is the algorithm for penetrating abdominal trauma applicable in emergency contexts?
Model evaluation	11	Were the algorithms overall clear in their writing and visual design?
Model evaluation	12	Do you consider the clinical relevance of the algorithms appropriate for your practice or training?
Model evaluation	13	Is the level of detail in the algorithms sufficient to guide diagnostic decisions?
Model evaluation	14	How would you rate the usability of the algorithms during clinical or academic practice?
Model evaluation	15	Do you find the algorithms complete, or do you identify important gaps in diagnostic processes?
Model evaluation	16	Do you perceive that the algorithms improve communication between requesting physicians and radiologists?
Model evaluation	17	Do you consider it feasible to integrate the algorithms into hospital workflows (emergency, inpatient, outpatient)?
Model evaluation	18	What level of educational usefulness do you attribute to the algorithms for students and residents in training?
Open feedback	19	Open-ended comments and suggestions

Data collection and analysis

Survey responses were collected digitally and stored in a centralized, encrypted database to ensure confidentiality. Quantitative data were analyzed using descriptive statistics, including frequency distributions and measures of central tendency. Likert-type responses were summarized as percentages and mean scores. Qualitative data from the open-ended question were analyzed using thematic content analysis. To strengthen the trustworthiness of the analysis, two independent researchers coded the responses and then discussed discrepancies until consensus was reached. This process enhanced interpretive consistency, which we describe as “reliability” in the qualitative analysis context. Synthesized results from both quantitative and qualitative strands were integrated to provide a comprehensive assessment of participant perceptions, highlighting strengths of the algorithms and identifying opportunities for improvement.

## Results

**Figure 1:**
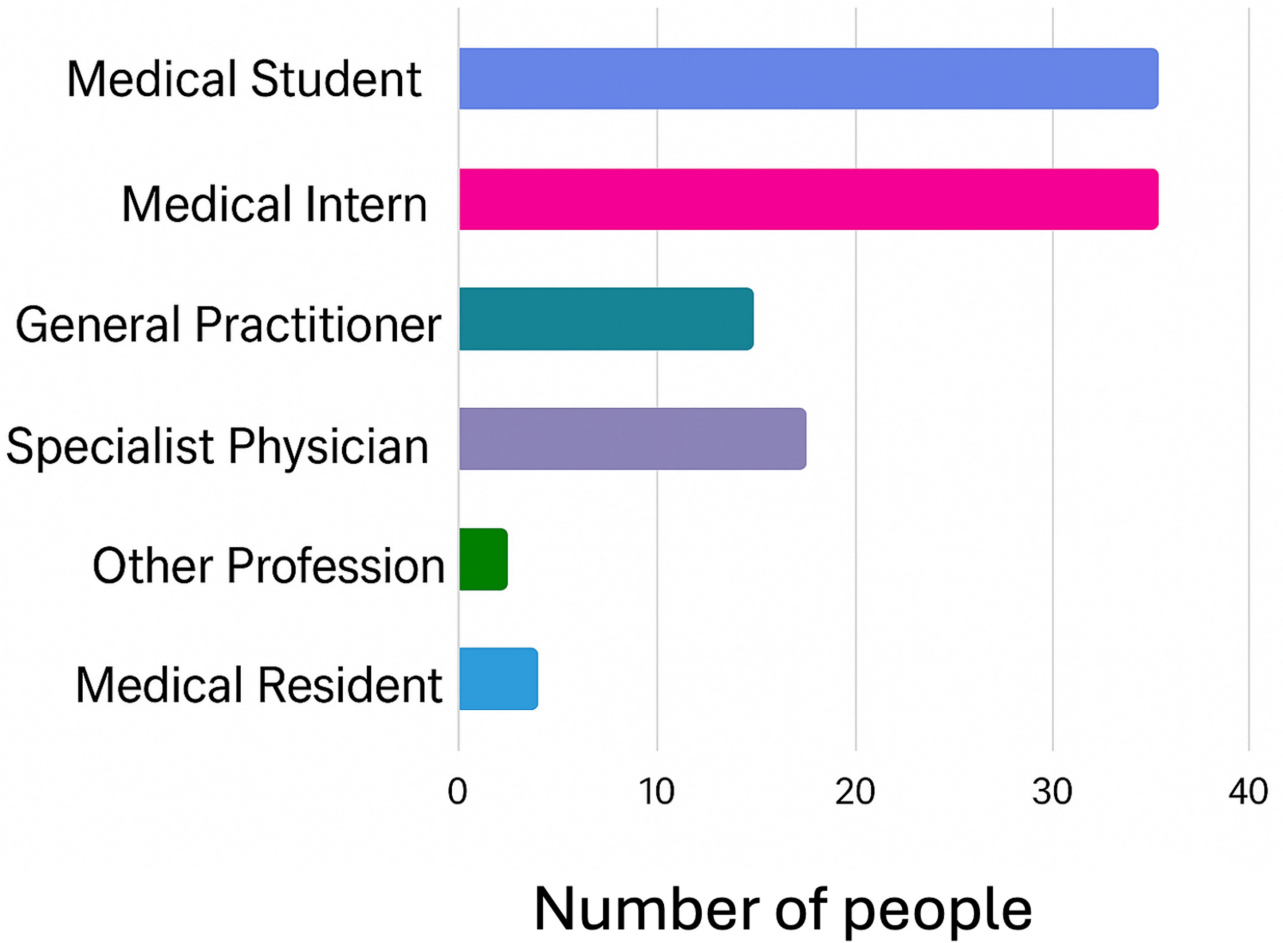
Bar chart showing the entire surveyed population by occupation variable

A total of 111 respondents participated, reflecting significant variability in their level of training. The sample included 36 medical students, 36 medical interns, 14 general practitioners, 17 medical specialists, five resident physicians, and three individuals from other professions. Additionally, three dentists (maxillofacial surgeons in training) were included; all had completed at least one year of clinical training. Dentists were included because, like medical trainees, they engage in clinical decision-making relevant to imaging studies (Figure 1).

Across the entire surveyed population, 100% (111 participants) responded positively regarding the overall usefulness of the algorithm, emphasizing its ability to improve diagnostic accuracy in complex diseases. Perceived usefulness was assessed using a Likert-type scale from 1 (not useful) to 5 (extremely useful), as described in the Methods section. This scale directly informed the results presented below. When asked to rate the importance of imaging study requests in radiology on a scale from 1 (low importance) to 5 (high importance), 98.2% (109 participants) assigned the highest rating, while 1.8% (2 participants) gave a score of 4. The average rating was 4.98 points. Radiology was the most frequently represented specialty (59%), followed by surgery and emergency medicine (12% each), and internal medicine (6%). Based on these results, all participants (100%) reported that the algorithm facilitates decision-making in abdominal emergencies (Table 2).

**Table 2:** Table showing the medical specialty of participants, including both practicing clinicians and physician administrators (i.e., trained medical specialists currently in administrative roles)

Specialty	Number of participants	% of total specialists (n=17)
Radiology	10	59%
Surgery	2	12%
Emergency medicine	2	12%
Internal medicine	1	6%
Other (administrative)	2	12%

**Figure 2:**
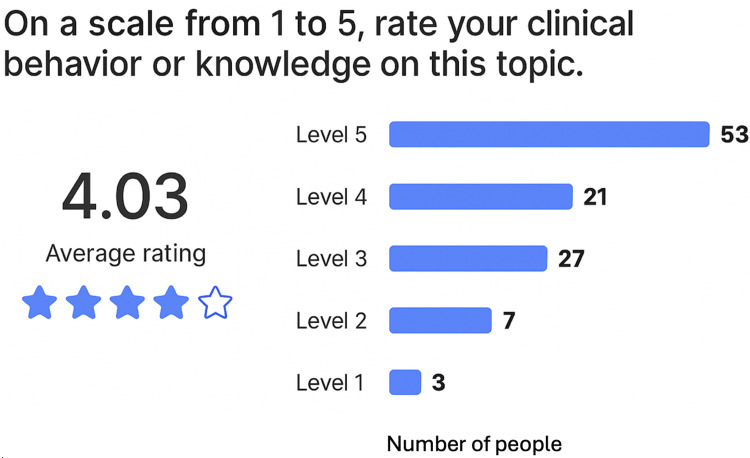
Bar chart representing the perceived level of knowledge regarding the request of imaging studies

Participants were also asked to assess their level of knowledge regarding imaging study requests using the same 1-5 scale. Results demonstrated considerable variability: 74 participants (66.7%) reported a high level of knowledge (levels 4-5), 27 participants (24.3%) reported an intermediate level (level 3), and 10 participants (9.0%) reported a low level (levels 1-2) (Figure 2).

Among the strengths identified, respondents highlighted the clarity of the algorithms for approaching patients according to their characteristics, as well as their practicality and ease of application in daily clinical practice. The main strength cited was the algorithm’s ability to reduce diagnostic delays and minimize errors in medical image interpretation.

**Figure 3:**
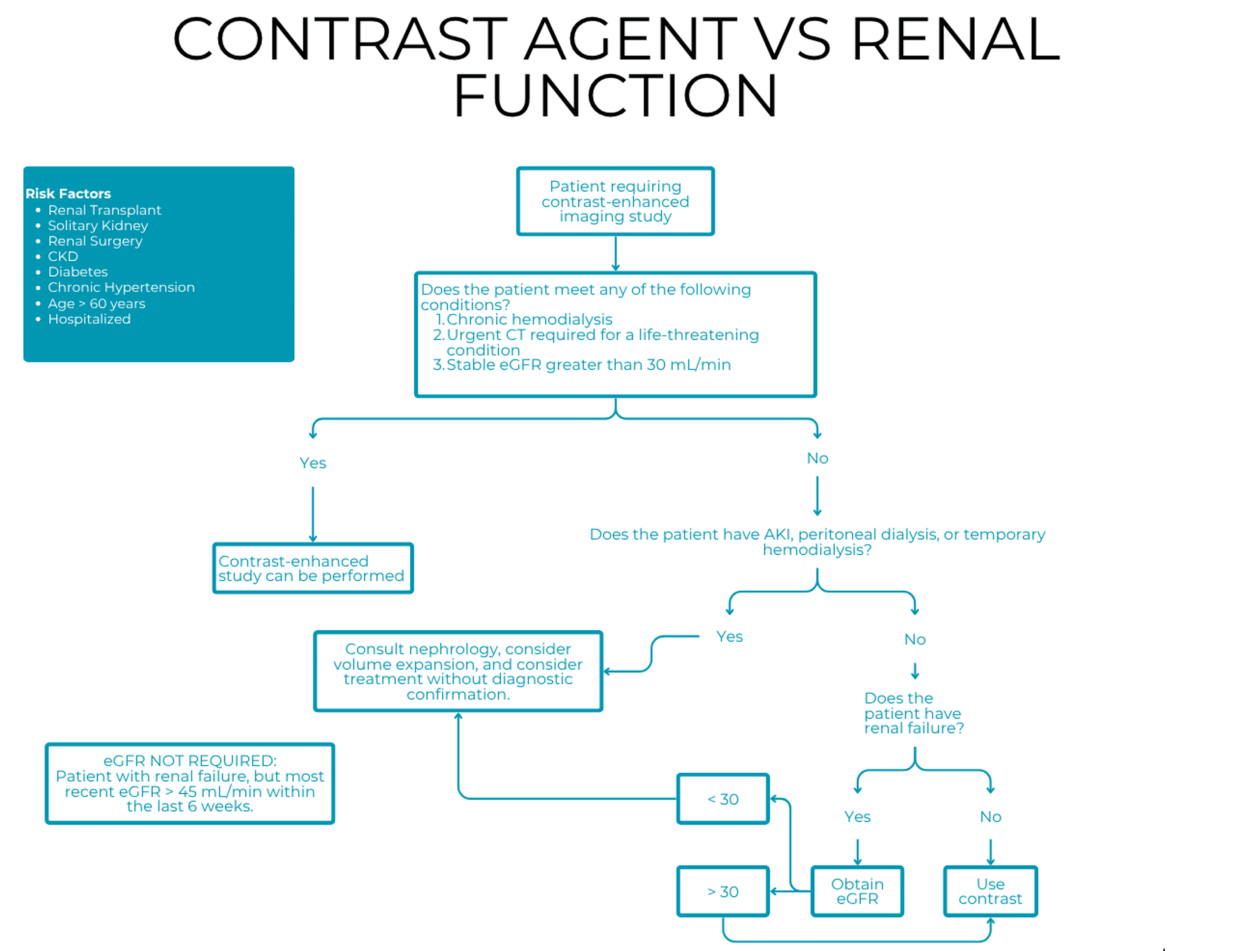
Algorithm for use of contrast agents in patients with or without renal function impairment who require contrast-enhanced imaging CKD: chronic kidney disease; CT: computed tomography; eGFR: estimated glomerular filtration rate

Another algorithm evaluated was designed to guide contrast media use in patients with or without renal impairment (Figure 3). This stepwise approach considers chronic kidney disease, acute kidney injury, and dialysis status, offering recommendations regarding whether to proceed with contrast-enhanced imaging, request serum creatinine, calculate estimated glomerular filtration rate (eGFR), or consult nephrology. The algorithm was adapted from institutional protocols and aligned with international guidelines (e.g., ESUR "European Society of Urogenital Radiology") [8]. Respondents noted its clarity, clinical applicability, and usefulness, emphasizing time optimization due to the rapid interpretation of its flowchart.

**Figure 4:**
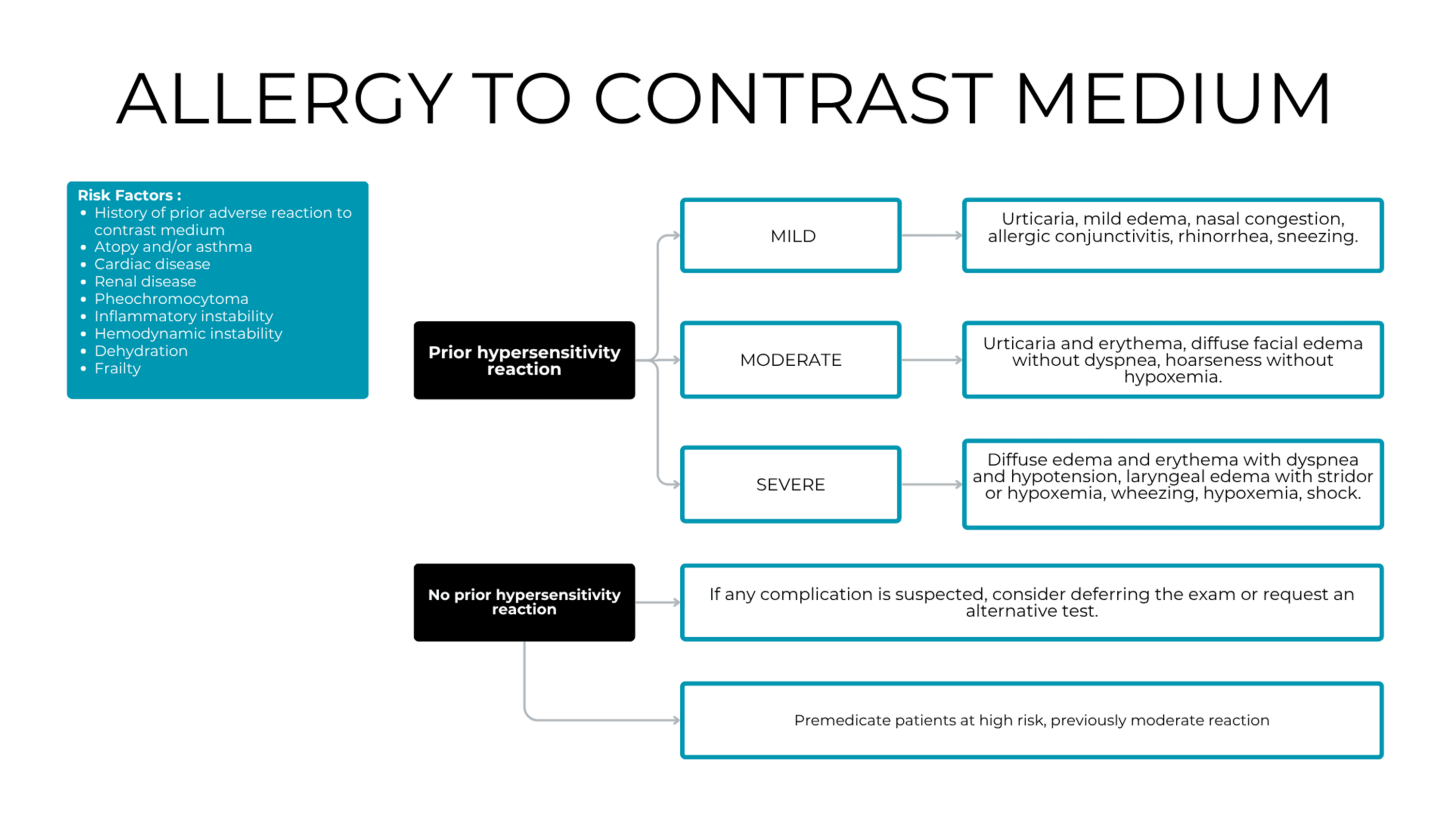
Classification of contrast media allergies

In the context of contrast media allergies, participants identified a knowledge gap. The algorithm incorporated risk factors to evaluate patients without prior hypersensitivity reactions and provided guidance for risk stratification and management. For patients with documented hypersensitivity reactions, specific recommendations were included according to the severity of the previous reaction (Figure 4).

Suggested improvements included the inclusion of typical clinical manifestations for each type of hypersensitivity reaction to prevent misclassification, and further clarification of causal relationships between symptoms and contrast administration [9,10].

The urinary tract infection algorithm, which addressed acute, recurrent, and relapsing infections [11,12], was well-received for its practicality and simplicity in supporting decision-making. Participants suggested improvements such as accounting for factors influencing urinary pH and providing greater detail regarding reinfection and recurrence outcomes.

**Figure 5:**
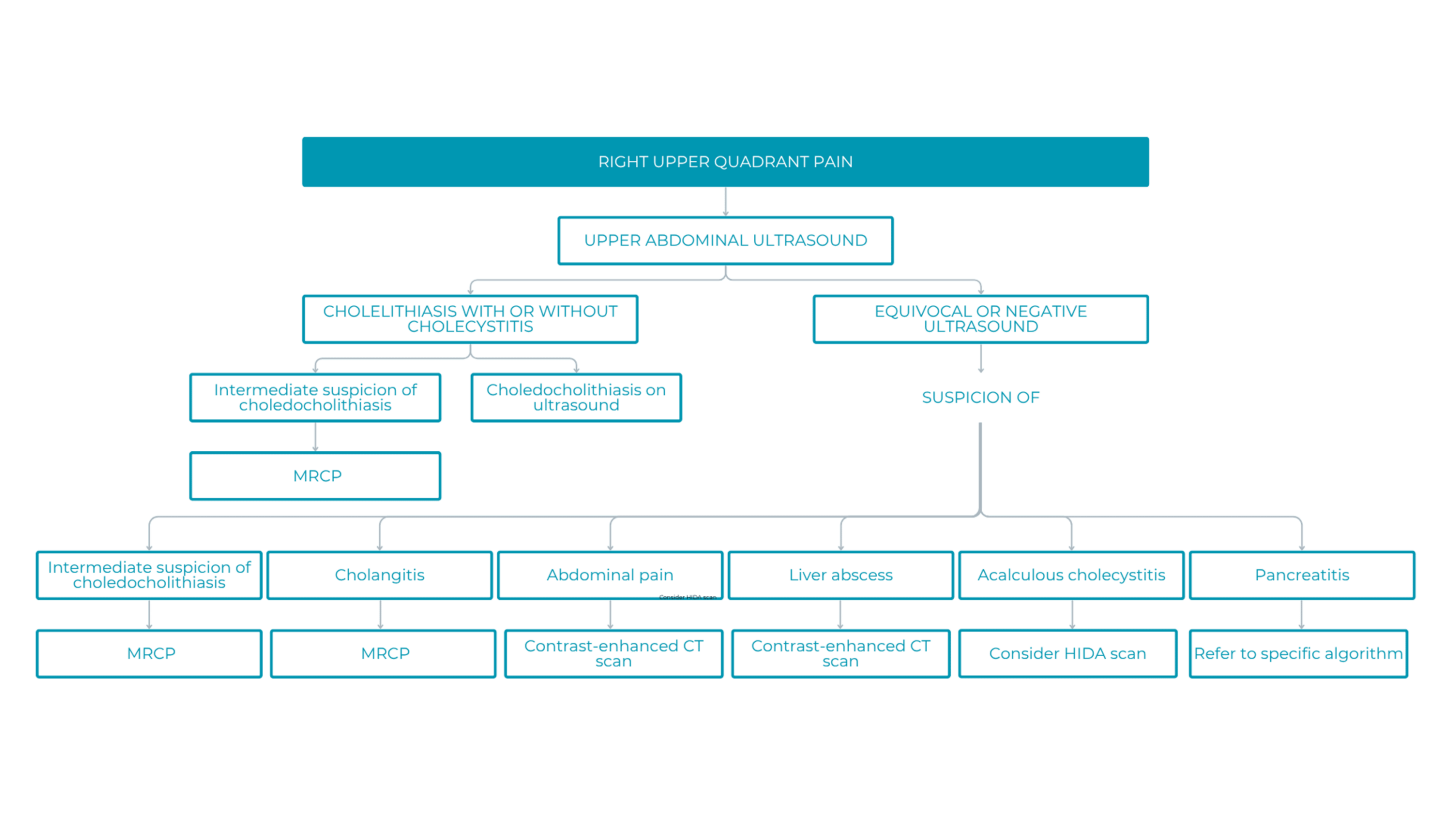
Algorithm for a patient with right upper quadrant abdominal pain MRCP: magnetic resonance cholangiopancreatography; CT: computed tomography; HIDA: hepatobiliary iminodiacetic acid scan

Right upper quadrant (RUQ) abdominal pain was another focus, particularly in acute care and emergency settings. RUQ pain accounts for approximately 5-10% of abdominal pain consultations in U.S. emergency departments, most often related to gallstones or acute cholecystitis [13-15]. The proposed algorithm emphasized abdominal ultrasound as the initial diagnostic step, followed by recommendations for subsequent imaging studies (Figure 5).

Respondents described this algorithm as excellent, clear, and highly practical. Limitations included the high cost and limited accessibility of certain modalities (e.g., MRCP and scintigraphy) and the lack of specification regarding the risk stratification scale for choledocholithiasis.

**Figure 6:**
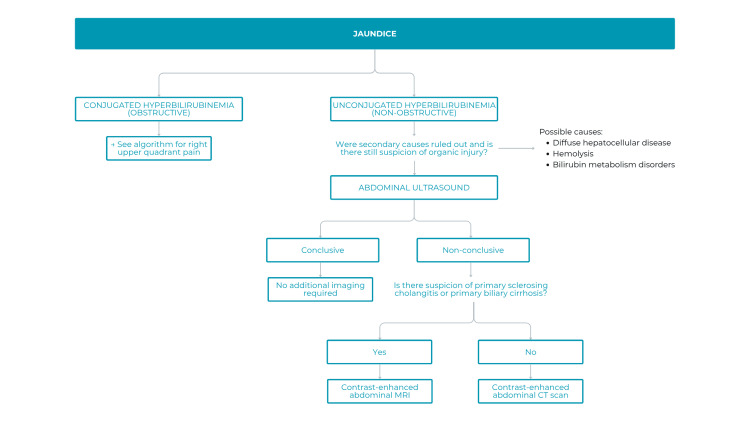
Algorithm for use in patients with jaundice MRI: magnetic resonance imaging; CT: computed tomography

Jaundice, frequently associated with RUQ pain, was also addressed, emphasizing differentiation between obstructive (direct hyperbilirubinemia) and non-obstructive causes (indirect hyperbilirubinemia), such as diffuse liver disease, hemolysis, or metabolic disorders [16,17]. The corresponding algorithm was positively received (Figure 6), highlighting clarity, structured approach, and practical guidance. Suggested improvements included more personalization by age and integration with related diagnostic algorithms.

The acute pancreatitis algorithm was valued for its simplicity, clarity, and patient-centered approach, particularly its inclusion of the time elapsed since symptom onset (72 hours), essential for assessing complications and guiding further studies [18]. Suggested improvements included incorporating additional causes and complications of pancreatitis and addressing barriers related to cost and access to diagnostic modalities.

**Figure 7:**
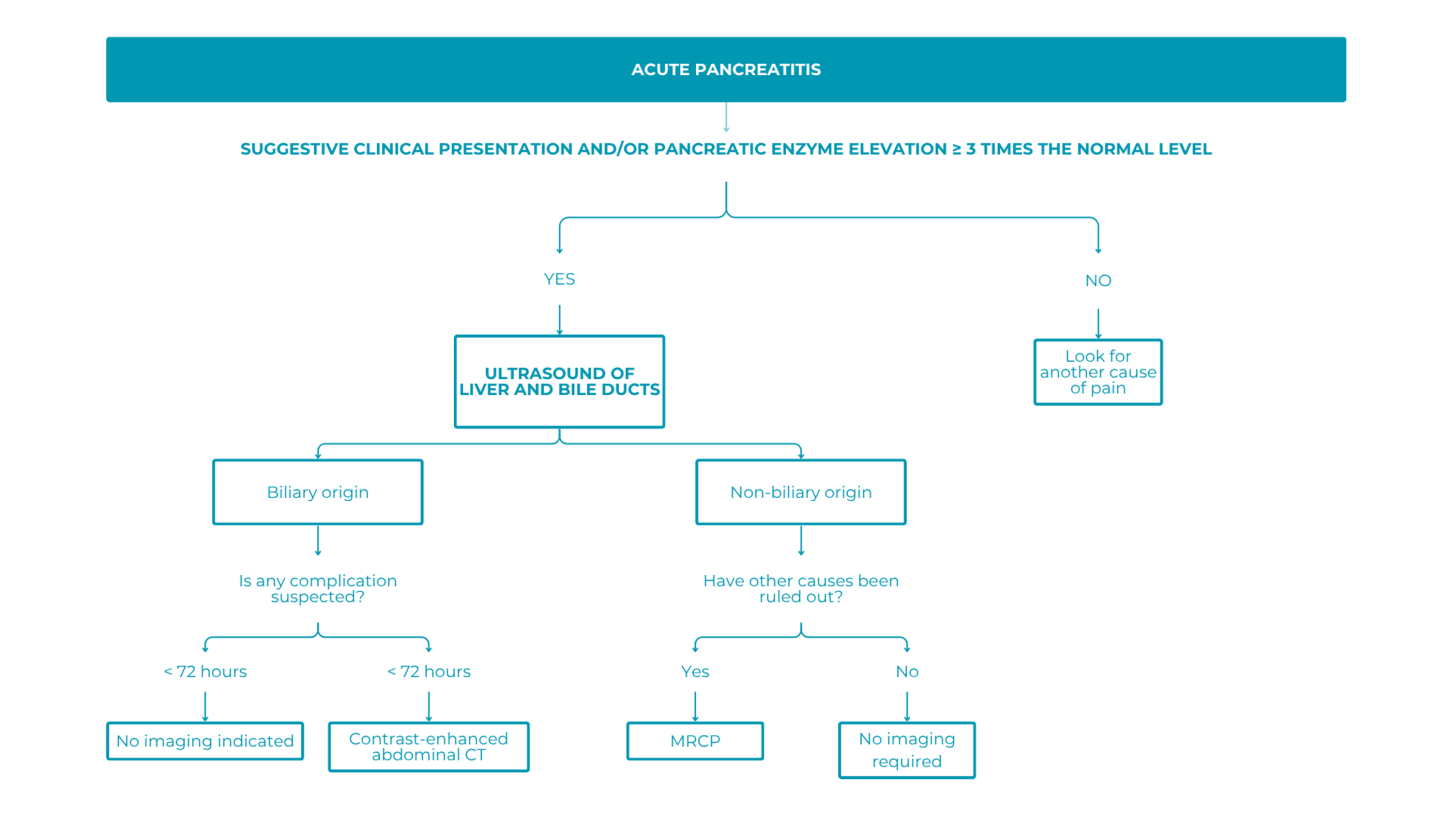
Algorithm for use in patients with acute pancreatitis CT: computed tomography; MRCP: magnetic resonance cholangiopancreatography

Overall feedback on the algorithms was highly positive, emphasizing clarity, practicality, and ease of use. Some suggested broadening coverage and addressing limitations in technological resources (Figure 7).

**Figure 8:**
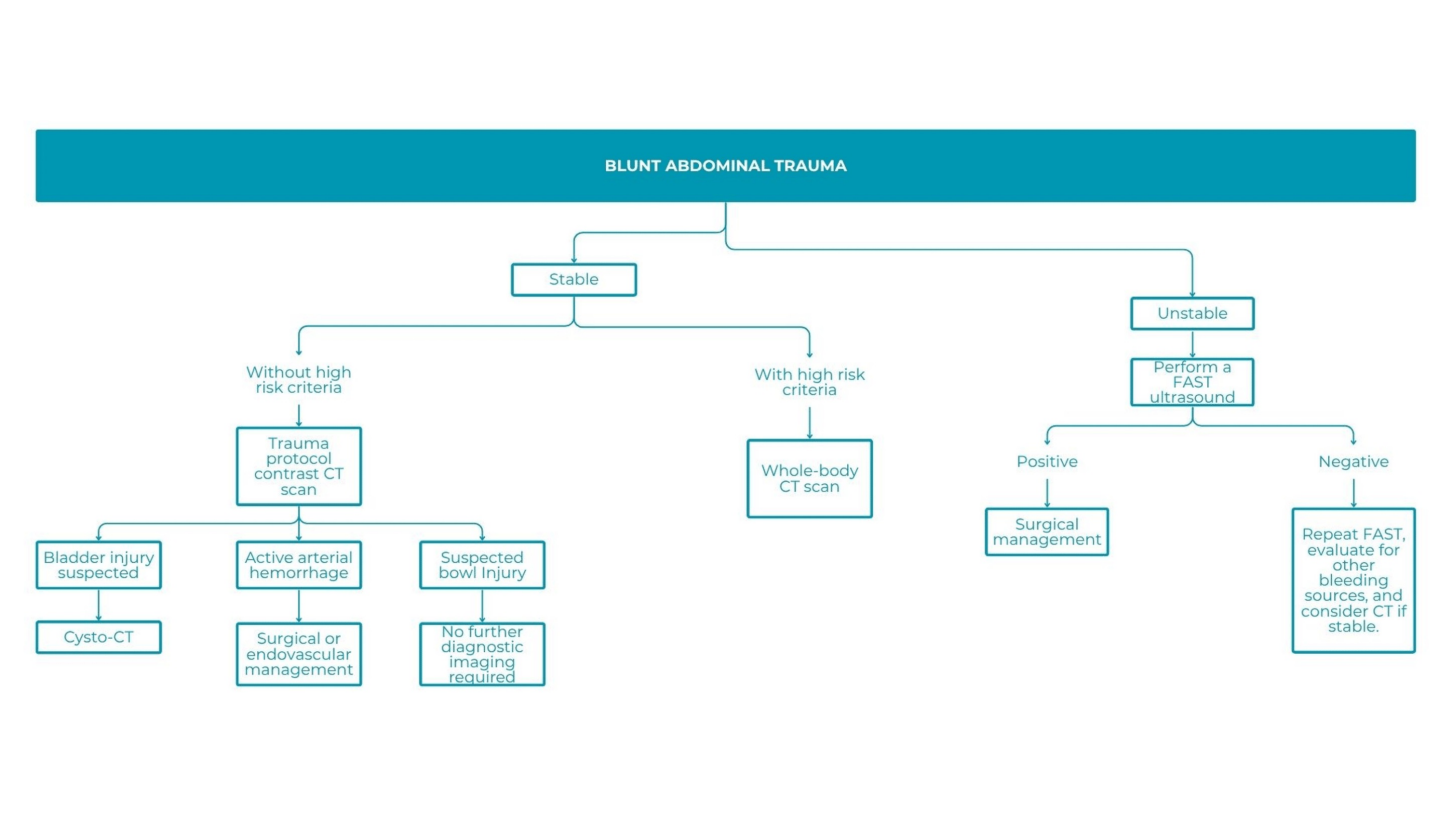
Algorithm for use in patients with blunt abdominal trauma CT: computed tomography; Cysto-CT: computed tomography-guided cystography; FAST: focused abdominal sonography for trauma

**Figure 9:**
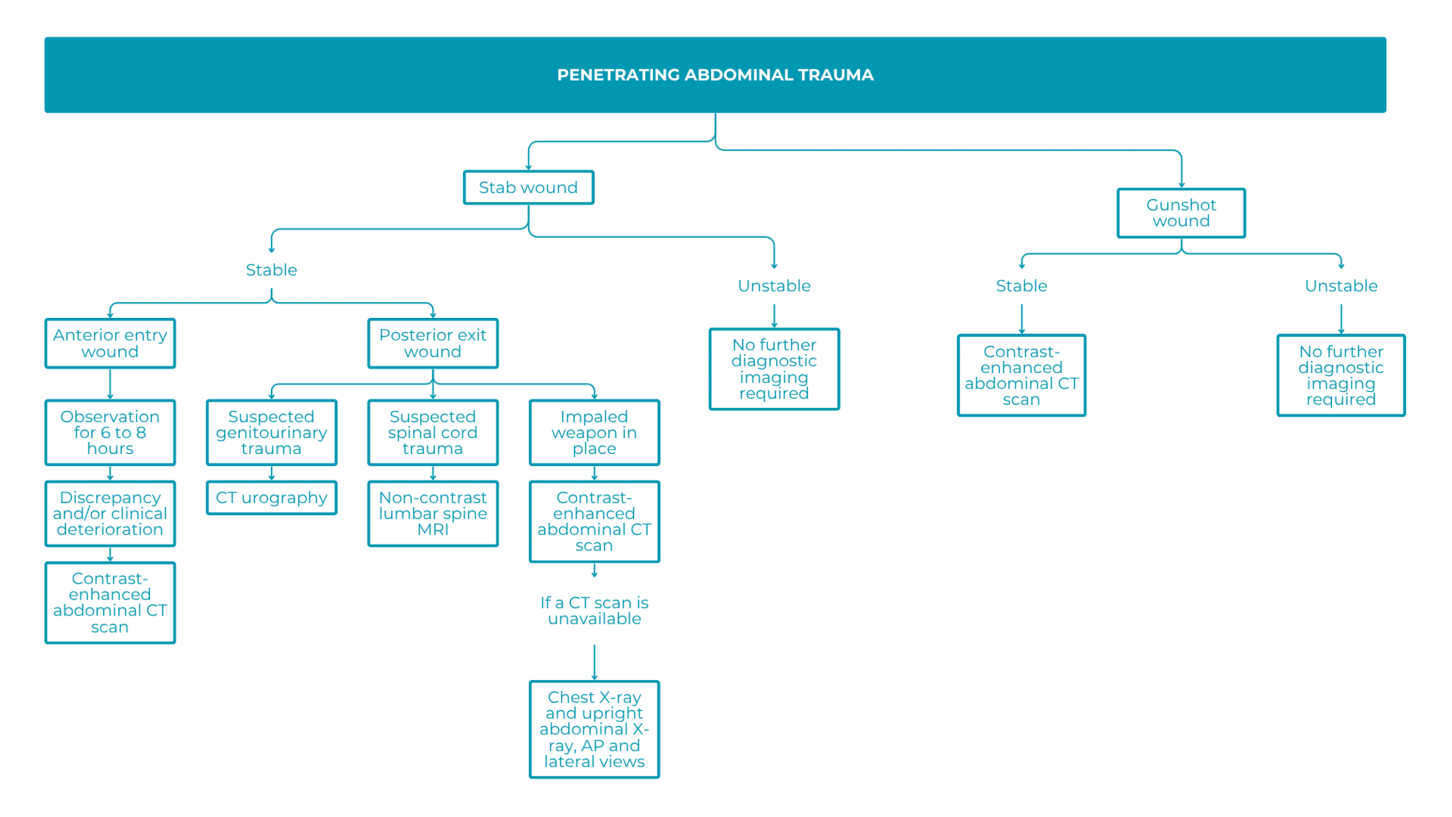
Algorithm for use in patients with penetrating abdominal trauma CT: computed tomography; MRI: magnetic resonance imaging; X-ray: radiography; AP: anteroposterior

Trauma algorithms were also evaluated. Polytrauma, involving at least two anatomical regions (e.g., head, chest, abdomen, pelvis, extremities), often requires rapid and comprehensive evaluation. CT was identified as the cornerstone of trauma assessment, typically including non-contrast imaging of the head and cervical spine, followed by contrast-enhanced imaging of the chest, abdomen, and pelvis, with nephrographic and excretory phases; some centers also add an arterial phase. Initial evaluations often include radiographs and FAST (Focused Assessment with Sonography for Trauma) (Figure 8), with MRI reserved for specific cases (Figure 9) [19].

According to responses from 53 participants, the advantages of the trauma algorithm included clarity, simplicity, and practicality, enabling structured and timely diagnosis. Suggested improvements included addressing operator dependency (especially with FAST), expanding focus to other potential etiologies (e.g., viral hepatitis), and enhancing personalization by patient age. Some noted the high cost and limited availability of whole-body CT, as well as the lack of detail regarding high-risk criteria and subsequent management steps.

In summary, while participants identified limitations related to resource availability, scope, and personalization, the overall feedback was strongly favorable. The algorithms were consistently described as clear, practical, and efficient tools supporting rapid and accurate decision-making across a range of clinical scenarios.

## Discussion

The survey results showed strong acceptance of the algorithms regarding their clinical utility, especially highlighting their ability to improve diagnostic accuracy and expedite decision-making in emergency situations. Most respondents considered that they facilitate diagnosis in complex pathologies such as abdominal pain, acute pancreatitis, and trauma, allowing for clear and rapid guidance on the request for radiological studies and the initial management of patients [20].

Constructive criticisms also arose, such as the operator dependence for performing certain tests like FAST ultrasound, the difficulty in applying some aspects of the algorithms in resource-limited settings, and the need to adjust them for different factors such as patient age or the availability of advanced technology like cholangioresonance. Regarding the pathologies analyzed, some participants suggested improvements to the imaging pathways, such as incorporating clearer guidance for evaluating jaundice in the context of suspected viral hepatitis or refining the imaging approach for identifying potential causes of pancreatitis. While definitive diagnosis of these conditions often involves clinical and laboratory assessment, participants expressed interest in tools that better integrate imaging within the broader diagnostic workflow [21].

The survey results show that the diagnostic algorithms have a high level of acceptance among medical students, trainees, radiologists, and healthcare administrators, who recognize their potential to improve efficiency and diagnostic accuracy. However, areas for improvement were also identified, such as the need for integration with existing hospital systems, reflecting a common challenge in implementing emerging technologies in clinical settings. Several previous studies have suggested that the adoption of technological tools in medicine largely depends on professionals’ perception, who must feel that these tools enhance their capabilities rather than replace their clinical judgment [22].

The proposed tool was considered viable by the surveyed participants, who emphasized the importance of integrating it into clinical workflows with minimal disruption. While some suggested its presence within the Picture Archiving and Communication System (PACS), integration at the point of order entry, such as within the Electronic Health Record (EHR), would be more effective for influencing imaging decisions [23].

The development of this tool has the potential to optimize the request for radiological studies in emergency settings. A similar framework could also be adapted to evaluate non-imaging workflows, supporting efficiency improvements across healthcare services.

Study limitations

This study provides insights for the early development and validation of decision-support tools in diagnostic imaging, particularly within academic and training environments. The participation of medical trainees and radiologists offered both educational and technical perspectives on the applicability of the proposed algorithms. Nonetheless, several limitations must be acknowledged. The sample was purposive and relatively small, which may limit the generalizability of the findings. To strengthen future research, it is important to clarify that participants were deliberately selected based on their active involvement in clinical decision-making and institutional guideline adoption, as well as their ability to provide targeted feedback for technical validation. The algorithm was evaluated in simulated rather than real clinical settings, so its practical impact remains untested. The tool has not yet been integrated with hospital information systems, which could pose challenges for implementation. Finally, although the qualitative feedback was valuable, it was analyzed descriptively without advanced statistical methods.

## Conclusions

The proposed algorithms were well received by the surveyed healthcare participants, who considered them useful for improving diagnostic accuracy and speeding up decision-making in emergency situations. Participants highlighted their effectiveness in facilitating decision-making for complex pathologies such as abdominal pain, acute pancreatitis, and trauma. The clarity, practicality, and ease of use of the algorithms were valued, making them useful tools in daily clinical practice. Despite the high acceptance, respondents suggested some improvements, such as the need to adjust the algorithms according to specific factors like patient age, availability of advanced technology, and accessibility to diagnostic resources (e.g., FAST ultrasound). Additionally, some aspects, such as the interpretation of contrast media allergies or the inclusion of more causes of liver diseases or pancreatitis, require greater clarity and depth.

In summary, the algorithms are promising tools with the potential to enhance clinical decision-making and efficiency in emergency management. Further refinements and integration into hospital systems, as well as adaptation to different clinical contexts and technological resources, will be essential for their successful implementation.

## References

[1] WHO Global Observatory for eHealth (2011). mHealth: new horizons for health through mobile technologies: second global survey on eHealth. World Health Organization.

[2] Davey S, Davey A (2014). Mobile-health technology: can it strengthen and improve public health systems of other developing countries as per Indian strategies? A systematic review of the literature. Int J Med Public Health.

[3] (2024). ACR Appropriateness Criteria®. https://www.acr.org/Clinical-Resources/Clinical-Tools-and-Reference/Appropriateness-Criteria.

[4] Sociedad Argentina de Radiología (2023). Guide of Recommendations for the Correct Request for Diagnostic Imaging Tests (In Spanish). Buenos Aires: Sociedad Argentina de Radiología.

[5] Walther F, Eberlein-Gonska M, Hoffmann RT, Schmitt J, Blum SF (2023). Measuring appropriateness of diagnostic imaging: a scoping review. Insights Imaging.

[6] Kim YW, Mansfield LT (2014). Fool me twice: delayed diagnoses in radiology with emphasis on perpetuated errors. AJR Am J Roentgenol.

[7] Lee CS, Nagy PG, Weaver SJ, Newman-Toker DE (2013). Cognitive and system factors contributing to diagnostic errors in radiology. AJR Am J Roentgenol.

[8] Thomsen HS (2006). European Society of Urogenital Radiology (ESUR) guidelines on the safe use of iodinated contrast media. Eur J Radiol.

[9] ACR Committee on Drugs and Contrast Media (2025). ACR Manual on Contrast Media. ACR Manual on Contrast Media.

[10] Shin J (2017). NSF vs. CIN: aggregated screening, safety, and protocol tools for contrast imaging in the setting of renal insufficiency. J Digit Imaging.

[11] Venkatesan AM, Oto A, Allen BC (2020). ACR Appropriateness Criteria® recurrent lower urinary tract infections in females. J Am Coll Radiol.

[12] Nikolaidis P, Dogra VS, Goldfarb S (2018). ACR Appropriateness Criteria(®) acute pyelonephritis. J Am Coll Radiol.

[13] Hogan S, Ward J, Sala E (2024). The utility of the abdominal series in the emergency setting: a retrospective review. Int J Emerg Med.

[14] Peterson CM, McNamara MM, Kamel IR (2019). ACR Appropriateness Criteria(®) right upper quadrant pain. J Am Coll Radiol.

[15] Yokoe M, Hata J, Takada T (2018). Tokyo Guidelines 2018: diagnostic criteria and severity grading of acute cholecystitis (with videos). J Hepatobiliary Pancreat Sci.

[16] Horowitz JM, Kamel IR, Arif-Tiwari H (2017). ACR Appropriateness Criteria(®) chronic liver disease. J Am Coll Radiol.

[17] Lalani T, Couto CA, Rosen MP (2013). ACR Appropriateness Criteria jaundice. J Am Coll Radiol.

[18] Porter KK, Zaheer A, Kamel IR (2019). ACR Appropriateness Criteria® acute pancreatitis. J Am Coll Radiol.

[19] Shyu JY, Khurana B, Soto JA (2020). ACR Appropriateness Criteria® major blunt trauma. J Am Coll Radiol.

[20] Pelaccia T, Sherbino J, Wyer P, Norman G (2025). Diagnostic reasoning and cognitive error in emergency medicine: Implications for teaching and learning. Acad Emerg Med.

[21] Davis DP, Campbell CJ, Poste JC, Ma G (2005). The association between operator confidence and accuracy of ultrasonography performed by novice emergency physicians. J Emerg Med.

[22] Zavala AM, Day GE, Plummer D, Bamford-Wade A (2018). Decision-making under pressure: medical errors in uncertain and dynamic environments. Aust Health Rev.

[23] Egoda Kapuralalage TN, Chan HF, Dulleck U, Hughes JA, Torgler B, Whyte S (2025). Clinical decision-making: Cognitive biases and heuristics in triage decisions in the emergency department. Am J Emerg Med.

